# Assessment tools measuring health-related empowerment in psychosocially vulnerable populations: a systematic review

**DOI:** 10.1186/s12939-021-01585-1

**Published:** 2021-11-17

**Authors:** Sandy Campbell, Jianxia Zhai, Jing-Yu Tan, Mursal Azami, Kym Cunningham, Sue Kruske

**Affiliations:** 1 College of Nursing & Midwifery, Charles Darwin University, Brisbane, Queensland Australia; 2 College of Nursing & Midwifery, Charles Darwin University, Melbourne, Victoria Australia; 3 College of Nursing & Midwifery, Charles Darwin University, Alice Springs, Northern Territory Australia

**Keywords:** Empowerment, Tools, Psychometric properties, Vulnerable populations, Systematic review

## Abstract

**Background:**

Many programs are undertaken to facilitate the empowerment of vulnerable populations across the world. However, an overview of appropriate empowerment measurements to evaluate such initiatives remains incomplete to date. This systematic review aims to describe and summarise psychometric properties, feasibility and clinical utility of the available tools for measuring empowerment in psychosocially vulnerable populations.

**Methods:**

A systematic literature review following the Preferred Reporting Items for Systematic Reviews and Meta-Analyses guidelines was completed. A descriptive approach was used for data analysis. Papers were eligible if they explored the development, validation, cross-cultural translation or the utility of an empowerment measurement tool in the context of psychosocially vulnerable populations.

**Results:**

Twenty-six included articles described twenty-six separate studies in which 16 empowerment measurement tools were developed, validated/translated, or used. There was heterogeneity in empowerment constructs, samples targeted, and psychometric properties measured. The measurement of reliability of the included instruments was satisfactory in most cases. However, the validity, responsiveness, interpretability, feasibility and clinical utility of the identified measurement tools were often not adequately described or measured.

**Conclusion:**

This systematic review provides a useful snapshot of the strengths as well as limitations of existing health related empowerment measurement tools used with psychosocially vulnerable populations in terms of their measurement properties, and constructs captured. It highlights significant gaps in empowerment tool measurement, development and evaluation processes. In particular, the results suggest that in addition to systematic assessments of psychometric properties, the inclusion of feasibility and clinical utility as outcome measures are important to assess relevance to clinical practice.

## Background

Empowerment of individuals refers to a participatory process of becoming stronger and more confident enabling them to have more control over their lives [1]. An empowered individual may display characteristics of increased self-esteem, self-efficacy, responsibility and self-determination [1]. However, the term empowerment has also been used with various populations and in a wide range of contexts to illustrate aspects of a broader concept [2]. As such it has been described as a multi-level construct, which comprises organisational, community or group and individual domains [3].

Empowerment has been viewed as a fundamental value or goal in health promotion and an integral element of social equity and social welfare policy [4, 5]. Empowerment-related research tends to identify and highlight participants’ strengths and abilities rather than focusing on risk factors and deficits [3]. Internationally, in varied health promotion programs researchers are endeavoring to conceptualise and measure empowerment, and aiming to inform theory building and policy advocacy [6–8].

In healthcare, vulnerable populations are those individuals at risk of unequal access to healthcare services and desirable health outcomes because they encounter barriers due to their cultural, ethnic, health or economic status [9]. Vulnerabilities can be further categorised into three domains: physical, psychological, and social [9]. Psychosocially vulnerable populations within the context of this review are characterised as those susceptible to poor health outcomes generated or exacerbated by the presence of particular psychosocial factors. Factors may include, but are not limited to, belonging to a racial or ethnic minority or being an indigenous person, being pregnant, a child, elderly or homeless, or having human immunodeficiency virus (HIV) or a severe mental illness. Psychosocially vulnerable populations are those at risk of disparate healthcare access and outcomes due to stigmatisation and prejudice [10]. Hence, empowerment that promotes independence and enables self-determination is often a goal for the holistic wellbeing of individuals from vulnerable populations [11].

Initiatives funded by WHO, USAID, the World Bank and other agencies, seek to build empowerment among vulnerable or disadvantaged groups and communities to eliminate stigma and health disparities [12]. Studies have shown that empowerment programs can lead to positive health-related outcomes such as improved coping skills, self-efficacy, self-mastery, more access to health services and other resources, and enable disadvantaged groups to drive positive structural and organisational change [13–17].

As the concept of empowerment has gained recognition as a core tenet in health promotion by patients, professionals, and policy makers, there has been increasing interest in the utility of implementing empowerment programs [18]. Endeavors to evaluate such interventions are largely dependent on effective and robust measurements of the empowerment concept [19]. However, to date, measurement has been complicated because there is no universally accepted definition of empowerment, and it is argued that the empowerment construct may be both context-dependent and population-specific [20].

Cyril et al. [4] stated that although there have been extensive studies on empowerment in the last decades, there remains a scarcity of literature adequately reporting on associated psychometrics. Whilst varied empowerment measurement tools and scales have been developed, their quality has not been rigorously or systematically appraised. Those studies that have appraised the reliability and validity of scales measuring empowerment have tended to focus on participants with specific diseases, limiting their generalisability to wider populations [21–23]. Because populations with psychosocial vulnerabilities tend to be at higher risk of social exclusion and reduced access to healthcare than the general population, it is important to determine the potential for well-measured empowerment interventions to be used in these groups.

To the best of our knowledge, there has been no published systematic review with regard to empowerment measurement tools available to evaluate and monitor benefits of health promotion programs for psychosocially vulnerable populations. Systematic examination of reliability, validity, feasibility and clinical utility of empowerment tools is required to inform the selection of appropriate instruments to evaluate empowerment programs and address outstanding issues on how to effectively enhance empowerment in individuals and groups. The purpose of the study was to systematically review and appraise the properties of empowerment measures and their applicability for use with empowerment programs for psychosocially vulnerable populations.

## Methods

### Search strategy

A systematic review was conducted according to the Preferred Reporting Items for Systematic Reviews and Meta-analysis (PRISMA) guidelines [24]. We searched MEDLINE, CINAHL, PsycINFO, PubMed, Informit Indigenous Collection, and the Australian Indigenous Health*InfoNet* electronic databases. The Australian database was included in addition to the international Informit Indigenous research resource collection because of the authors’ awareness of ongoing Australian-based research about empowerment assessment in Aboriginal and Torres Strait Islander communities. The searches used relevant Medical Subject Headings (MeSH) and keywords listed below (Pubmed example). To identify additional eligible studies that may have been missed by the electronic search, the reference lists of the retrieved articles were also reviewed, supplemented by citation tracking using Google scholar. Papers published between January 1990 and January 2021 were eligible for inclusion. The database search inception date of January 1990 was selected because the publication of health-related empowerment studies has increased dramatically since the early 1990s [1]. We conducted the database searches for the review between 4 December 2020 and 31 January 2021. Retrieved literature from the combined database searches was imported into bibliographic citation management software, Endnote X9.

**Table:** 

#1	empowerment [MeSH Terms]
#2	empowerment measurement* [Title/Abstract]
#3	empowerment scale*[Title/Abstract]
#4	empowerment tool*[Title/Abstract]
#5	empowerment survey*[Title/Abstract]
#6	empowerment questionnaires*[Title/Abstract]
#7	#1OR #2 OR #3OR #4 OR #5 OR #6
#8	vulnerable population* [MeSH Terms]
#9	sensitive population* [Title/Abstract]
#10	underserved population* [Title/Abstract]
#11	#8 OR #9 OR #10
#12	#7 AND #11

### Study inclusion and exclusion criteria

Articles were included if the study aims focused on empowerment measurement tool development, or the implementation, validation or translation of existing empowerment measurement tools in the context of psychosocially vulnerable populations. Studies investigating empowerment as a health outcome measure to evaluate the utility of empowerment measurement tools contextualised with psychosocially vulnerable individuals were also eligible. Only articles available in English language were included. There were no restrictions on study quality. Studies that were published in dissertations, books, reports, and other non-peer-reviewed resources were also eligible for inclusion. Studies were excluded if empowerment was explored using only qualitative research methods (e.g. face to face interviews or focus groups), they did not focus on empowerment in a health-related context, or they did not report any psychometric assessment results from measuring empowerment.

### Data extraction and data items

Data extraction comprised general information about the study including author, year, study design, setting and study aims, and participant characteristics. We extracted further detailed information with regard to characteristics of empowerment measurement tools, the empowerment domains under examination, measurement tool item development, number of items included in each tool, how the measurement tool was administered, tool response scales, and whether exploratory factor analysis (EFA) and/or confirmatory factor analysis (CFA) was conducted.

We adhered to the guidelines for instrument measurement properties suggested by Rostad et al. [25]. The psychometric properties of the empowerment measurement tools were appraised across four dimensions: reliability, validity, responsiveness and interpretability. In addition, we appraised feasibility and clinical utility of the tools. In this review, reliability refers to the consistency of a measurement, which usually includes test-retest reliability, internal consistency, and inter-rater reliability [26]. Validity refers to the extent to which a measurement tool represents the variable/s it is intended to measure [26]. Responsiveness reflects the capacity of an instrument to measure change over time, and interpretability of measurement scores is important to differentiate between clinically important change and measurement error [27]. Feasibility refers to the resources needed to administer and process a participant assessment using the measurement tool, for example, who completed the assessment, time taken, and amount of staff training required [25, 28]. Clinical utility explores ‘usefulness to practice’ and whether the result of the assessment can inform clinical and industry decision making [29].

### Data synthesis and presentation

A descriptive analysis was utilised in this study to illustrate the range of empowerment measurement tools used with psychosocially vulnerable populations, and evaluate their psychometric properties, feasibility of use and clinical utility. The study results were tabulated and presented using descriptive summaries.

## Results

### Included studies

**Fig. 1:**
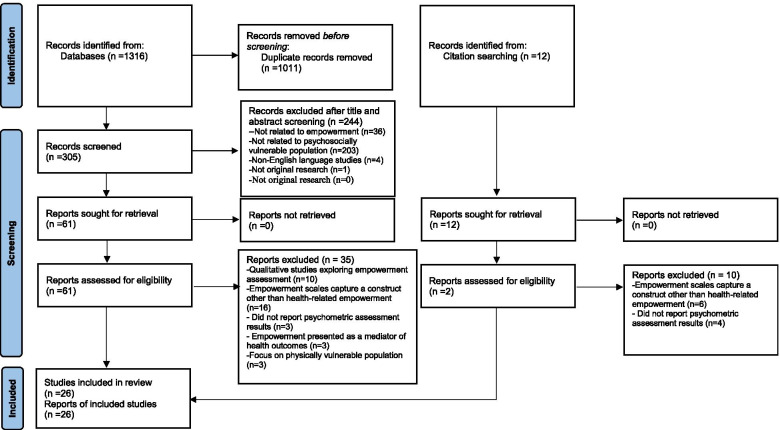
PRISMA flow diagram of study selection. *Adapted from:* Page MJ, McKenzie JE, Bossuyt PM, Boutron I, Hoffmann TC, et al. The PRISMA 2020 statement: an updated guideline for reporting systematic reviews. BMJ 2021;372:n71

Electronic searches yielded 1316 articles and the secondary reference list search generated 12 additional papers (Fig. 1). After removing 1011 duplicate publications, 305 records remained for title and abstract review. Screening of titles and abstracts excluded 244 papers. The remaining 61 full-text records were reviewed for inclusion eligibility. A further 35 articles were excluded. There was final inclusion of 26 papers focusing on empowerment measurement tool development, or the validation, translation or application of existing empowerment measurement tools.

### Overview of the studies

In total, the 26 included articles reported 26 distinct studies and 16 different empowerment measurement tools (Table 1). Eight of the studies were undertaken in the US, five in Australia, two in India, and two in Japan. One study was in both the US and Australia, and one each were undertaken in Nepal, Iran, the Netherlands, China, Mexico, Bolivia, Sweden and Africa. With regard to the empowerment measurement tools, the number of response items included in the tools ranged from eight to 34. A majority of the studies used a measurement tool with a four or five-point Likert scale. Study sample sizes ranged from 15 to 1824 participants. Characteristics indicating psychosocial vulnerability among study participants included pregnancy, mental health disorders (including families of children with mental health disorders), Indigenous populations, ethnic minorities, people infected with HIV, and people who were members of self-help groups. Across the 26 included studies, seven articles focused on initial development of an empowerment measurement tool (tool development studies), five articles reported how the tools were validated or translated when used in a cross-cultural or new language setting (tool validation/translation study), and the remaining 14 articles used an empowerment measurement tool to assess health outcomes following an intervention (empowerment study).

**Table 1:** Characteristics of the empowerment measurement adopted in the review

Author (year)	Country	Study aim	Participants characteristics	Measure	Item development	No. of scale items	Methods of administration	Domains of empowerment	Response scale	EFA and/ or CFA
Anderson, Funnell [30]	USA	To evaluate the effectiveness of a problem-based empowerment patient education program targeting urban African Americans with type 2 diabetes	*N* = 239 Mean age = 61 Mean years since diagnosis = 8.5 Currently Married = 31% Completed high school = 73% Without insurance = 73%	Diabetes Empowerment Scale Short-Form (DESSF)	NR	8	Self-administered	8 domains: 1) assessing the need for change 2) developing a plan 3) overcoming barriers 4) asking for support 5) supporting oneself 6) coping with emotion 7) motivating oneself; 8) making diabetes care choices appropriate for one’s priorities and circumstances	4-Point Likert Scale	NR
Bhatta and Liabsuetrakul [31]	Nepal	To assess effectiveness of an empowerment intervention to HIV infected people receiving prevention and antiretroviral therapy	*N* = 132 Mean age (intervention group) = 36.3 Mean age (control group) = 35.8 The majority had a low family income, was married and had children	Empowerment Scale	NR	28	Self-administered	5 domains: 1)self-efficacy/self-esteem 2)power-powerlessness 3) community activism and autonomy 4) optimism and control over the future 5) righteous anger	5-Point Likert Scale	NR
Blanchard, Mohan [15]	India	To assess effectiveness of empowerment program for HIV prevention among female sex workers	*N* = 1750 Mean age = 32, majority were unable to read or write	Empowerment survey	Adapted from previous surveys	NR	interviewer-administered	3 domains: 1) power with: a sense of individual self-esteem and confidence 2) power within: collective identity and solidarity 3) power over: reflects access to social entitlements	4-Point Likert Scale	EFA
Borghei, Taghipour [32]	Iran	To development and validation of a new tool to measure Iranian pregnant women’s empowerment	*N* = 161 Mean age = 25.8 A great majority of pregnant mothers (92.5%) were primigravidae and most of them (87.0%) lived independently with their husbands	Self-Structured Pregnancy Empowerment Questionnaire	Literature review, panel consultation and pilot testing	32	Self-administered	3 domains: 1) educational empowerment 2) autonomy 3)socio-political empowerment	4-Point Likert Scale	EFA
Cheung, Mok [33]	Hongkong, China	To examines the relationship between personal empowerment and life satisfaction among self-help group members	*N* = 719 31 to 40 years old (27.1%) 41 to 50 years old (30.2%) 51 to 60 years old (16.1%) Above 61 year old (19.4%) majority had a rather low education level	Personal empowerment Scale	NR	20	Self-administered	3 domains: 1) intrapersonal empowerment 2) interpersonal empowerment 3) extrapersonal empowerment	6-Point Likert Scale	NR
Contreras-Yáñez, Ruiz-Medrano [20]	Mexico	To adapt the Spanish version of the Health Empowerment Scale (S-HES) in RA patients from Latin American	*N* = 270 Patients were primarily middle-aged females, married, had basic education and medium-low socioeconomic status	RA Empowerment Scale for Hispanic patients (RAEH)	Literature review, panel consultation and pilot testing	8	Self-administered	8 domains: 1) satisfaction and dissatisfaction related to health 2) identification and achievement of personally meaningful goals 3) application of a systematic problem-solving process 4) coping with the emotional aspects of living with health 5) stress management 6) appropriate social support 7) self-motivation 8) making cost/benefit decisions about making behavior changes	5-Point Likert Scale	EFA
Corrigan [34]	USA	To assess relationship between participation in consumer operated services and measures of recovery and empowerment in people with psychiatric disability	*N* = 1824 1094 were women (60%) Mean ± SD = 41.8 ± 10.4 1356 (74%) was European American	Empowerment Scale	NR	28	Self-administered	5 domains: 1)self-efficacy/self-esteem 2)power-powerlessness 3) community activism and autonomy 4) optimism and control over the future 5) righteous anger	4-Point Likert Scale	NR
Dempsey and Dunst [35]	USA, and Australia	To investigate how help-giving practices operate to produce variations in family empowerment	*N* = 141 Most respondents were mothers; Majority of Children of Australian respondents were more likely to be over 3 years of age	Family Empowerment Scale (FES)	NR	34	Self-administered	2 domains: 1) level of empowerment (individual, service and community) 2) expression of empowerment (attitude, knowledge and behaviour)	NR	EFA
Diamond-Smith, Treleaven [36]	India	To explore whether measures of women’s empowerment are associated with their experiences of mistreatment at their last childbirth	*N* = 759 young women aged 16–30 living in slum areas; All women had given birth in the last 5 years	Gender Equitable Men scale	NR	24	Self-administered	4 domains: 1)violence 2) sexual relationships 3) reproductive health and disease prevention 4) domestic chores and daily life	3-Point Likert Scale	NR
Farber and Maharaj [37]	USA	To evaluate effectiveness of a group-based education curriculum empowerment program on high-risk African American families with children with developmental delays	*N* = 39 32 participants (82%) aged 30–49 Participants had on average about three children listed as living at home and under 18 years of age	Shortened Family Empowerment Scale (FES)	NR	16	Self-administered	2 domains: 1) level of empowerment (individual, service and community) 2) expression of empowerment (attitude, knowledge and behaviour)	5-Point Likert Scale	NR
Godoy, Patel [38]	Bolivia	To explore nutritional status and spousal empowerment among native Amazonians	*N* = 440 Of the 231 households, 209 households were headed jointly by a wife and by a husband, and 22 were headed by a single adult	Individual empowerment survey	Literature review	10	Self-administered; Cross-checking	2 domains: 1)Decider 2) tie breaker	5-Point Likert Scale	NR
Hansson and Björkman [39]	Sweden	To assess reliability and validity of the Swedish version of an empowerment scale in people with a mental illness	*N* = 92 Mean age = 47 Approximately 60% of the subjects had a schizophrenia diagnosis and a further 20% other psychosis diagnoses	Making Decisions scale	Adaption from the original empowerment scale (ES)	28	Self-administered	5 domains: 1)self-efficacy/self-esteem 2)power-powerlessness 3) community activism and autonomy 4) optimism and control over the future 5) righteous anger	4-Point Likert Scale	EFA
Haswell, Kavanagh [17]	Australia	To validate psychometric properties of the Growth and Empowerment Measure (GEM) in Indigenous Australians	*N* = 184 Mean age = 39.9 163 Aboriginal (88.6%), 13 Torres Strait Islander (7.1%) or both (4.3%)	Growth and Empowerment Measure (GEM)	Literature review, panel consultation and pilot testing	14-item and 12 Scenarios	Self-administered	2 domains: 1) Emotional Empowerment Scale (EES) (Self-Capacity; Inner Peace) 2) 12S (Healing and Enabling Growth, Connection and Purpose)	EES:5-Point Likert Scale 12S: 7-Point Likert Scale	EFA
Homko, Sivan [40]	USA	To examine the effect of self-monitoring blood glucose on feelings of self-efficacy in women with gestational diabetics	*N* = 58 Maternal mean age (mean ± SD) SMBG group: =30.3 ± 5.4 PM group: 29.0 ± 6.4	Diabetes Empowerment scale	NR	23	Self-administered	5 domains: 1) setting goals 2) solving problems 3) obtaining support 4) motivating oneself 5) making decisions	5-Point Likert Scale	NR
Jersky, Titmuss [13]	Australia	To evaluate effectiveness an urban art-based community health program on improving health service access and wellbeing of young Aboriginal parents	*N* = 92 88 females, 4 males	Growth and Empowerment Measure (GEM)	NR	14-item and 10 Scenarios	Self-administered	2 domains: 1) Emotional Empowerment Scale (EES) (Self-Capacity; Inner Peace) 2) 10S (Healing and Enabling Growth, Connection and Purpose)	EES:5-Point Likert Scale 12S: 7-Point Likert Scale	NR
Kaczinski, Rosenheck [41]	USA	To assess psychometric property of empowerment and confidence among veterans with psychiatric disabilities	*N* = 296 Mean age = 48.5; Majority participants male (95%) and white (66%)	Empowerment Scale	NR	28	Self-administered	5 domains: 1)self-efficacy/self-esteem 2)power-powerlessness 3) community activism and autonomy 4) optimism and control over the future 5) righteous anger	4-Point Likert Scale	NR
Kameda and Shimada [42]	Japan	To develop an empowerment scale for pregnant women	*N* = 171 Mean age (Mean ± SD) = 29.1 ± 4.4; gestational age = 27.8 ± 9.8	Empowerment Scale for pregnant women	Literature review, panel consultation and pilot testing	27	Self-administered	5 domains: 1)self-efficacy 2) future image 3)self-esteem 4) support and assurance from others 5) joy of an addition to the family	4-Point Likert Scale	EFA
Klima, Vonderheid [43]	USA	To develop a Pregnancy-related Empowerment Scale and adapted in Spanish-speaking population	*N* = 365 Mean age (mean ± SD) = 27.1 ± 6.40 36.1% were Black non-Hispanic, 42.6% were White non-Hispanic and 21.3% were Hispanic	Pregnancy-Related Empowerment Sscale (PRES)	Literature review, panel consultation and pilot testing	16	Self-administered	4 domains: 1) provider connectedness 2) skilful decision-making 3) peer Connectedness 4) gaining voice	4-Point Likert Scale	CFA
Koren, DeChillo [44]	USA	To measure empowerment in families with children having emotional disabilities	N = 440 Mean age (mean ± SD) = 40 ± 6.6 Majority are female, white	Family Empowerment Scale (FES)	Literature review, pilot testing	34	Self-administered	3 domains: 1)Family 2) service system 3)community/political	5-Point Likert Scale	EFA
LoGiudice, Josif [45]	Australia	To describe demographic features and wellbeing of carers of Aboriginal Australians	*N* = 124 Mean age (mean ± SD) =38.8 ± 15.0 majority (97.6%) identified as Aboriginal	Growth and Empowerment Measure (GEM)	NR	14-item and A 6 item short form (Core6)	Self-administered	2 domains: 1) Emotional Empowerment Scale (EES) (Self-Capacity; Inner Peace) 2) Core 6 (Healing and Enabling Growth, Connection and Purpose)	EES:5-Point Likert Scale 12S: 7-Point Likert Scale	NR
Patil, Klima [14]	Africa	To investigate how antenatal care affects aspects of women’s sense of control over their pregnancy	*N* = 218 All participants aged 16 years old, and 20–24 weeks pregnant	Pregnancy-Related Empowerment Scale (PRES)	NR	16	Self-administered	4 domains: 1) provider connectedness 2) skilful decision-making 3) peer Connectedness 4) gaining voice	4-Point Likert Scale	NR
Nishita, Cardazone [46]	USA	To examine effectiveness of empowerment program: life coaching and pharmacist counseling for employed adults with diabetes	*N* = 190 Mean age (mean ± SD) =48.46 ± 0.71 6% of whom were Asian and 35% of whom were Native Hawaiian or Pacific Islander	Diabetes Empowerment Scale Short-Form (DESSF)	NR	8	Self-administered	8 domains: 1) assessing the need for change 2) developing a plan 3) overcoming barriers 4) asking for support 5) supporting oneself 6) coping with emotion 7) motivating oneself; 8) making diabetes care choices appropriate for one’s priorities and circumstances	4-Point Likert Scale	NR
Yamada and Suzuki [47]	Japan	To assess the levels of empowerment in Japanese patients with chronic schizophrenia	*N* = 72 Mean age (mean ± SD) =41.7 ± 10.7, men, *n* = 43; women, *n* = 29	Empowerment Scale-J		28	Self-administered	5 domains: 1)self-efficacy/self-esteem 2)power-powerlessness 3) community activism and autonomy 4) optimism and control over the future 5) righteous anger	4-Point Likert Scale	NR
Castelein, van der Gaag [48]	Netherlands	To compare three instruments that are used to measure empowerment of people with psychotic disorders	*N* = 50 Mean age (mean ± SD) =31.4 ± 13.0 mean duration of illness was 6.5 ± 6.3 years The diagnostic criteria for 39 participants were diagnosed with schizophrenia and 11 had a related psychotic disorder	Empowerment Scale (ES); the Personal Empowerment Scale (PES); the Mental Health Confidence Scale (MHCS)	NR	ES: 28 PES: 20 MHCS: 16	Self-administered	ES 5 domains: 1)self-efficacy/self-esteem 2)power-powerlessness 3) community activism and autonomy 4) optimism and control over the future 5) righteous anger PES 2 domains: 1)discretion 2) reduction in chance MHCS 3 domains: 1)optimism 2) coping 3) advocacy	NR	NR
Bovill, Bar-Zeev [49]	Australia	To pilot the Growth and Empowerment Measure (GEM) with a sample of pregnant Aboriginal women who smoke	*N* = 15 Mean age = 27.2 ± 5.5 and the average duration of pregnancy in weeks was 19.2	Growth and Empowerment Measure (GEM)	NR	14-item and a 6 item short form (Core6)	Self-administered	2 domains: 1) Emotional Empowerment Scale (EES) (Self-Capacity; Inner Peace) 2) Core 6 (Healing and Enabling Growth, Connection and Purpose)	EES:5-Point Likert Scale 12S: 7-Point Likert Scale	NR
Berry, Crowe [50]	Australia	To examines the sensitivity to change of the new Growth and Empowerment Measure (GEM) for Indigenous Australians in Substance Abuse Treatment	*N* = 103 included 57 Indigenous and 46 non-Indigenous males over 18 years of age	Growth and Empowerment Measure (GEM)	NR	14-item and A 6 item short form (Core6)	Self-administered	2domains: 1) Emotional Empowerment Scale (EES) (Self-Capacity; Inner Peace) 2) Core 6 (Healing and Enabling Growth, Connection and Purpose)	EES:5-Point Likert Scale 12S: 7-Point Likert Scale	NR

*EFA* exploratory factor analysis, *CFA* confirmatory factor analysis, *NR* Not reported

### Measurement properties

#### Reliability

Internal consistency of the empowerment measurement tools was tested in 18 studies and most demonstrated medium to moderately good internal consistency across settings (Table 2) with three reporting poor internal consistency of sub-scales [39, 42, 48]. Test-retest reliability was assessed in four studies [20, 40, 41, 44]. Only one study by Contreras-Yáñez et al. [20] reported intra-class coefficients (ICC). The study assessed adaption of a Spanish version of the Health Empowerment Scale for use with Latin American participants with rheumatoid arthritis and the ICC showed moderately good reliability across settings.

**Table 2:** Measurement properties of the scales included in the review

Study Author (year)	Reliability		Validity				Responsiveness	Interpretability
	Internal consistency (Cronbach’s alpha)	Test–retest reliability	Content validity	Face validity	Criterion validity	Construct validity		
Anderson, Funnell [30]	Cronbach α = 0.84(total scale)	NR	NR	NR	NR	NR	NR	NR
Bhatta and Liabsuetrakul [31]	Cronbach α = 0.97 (total scale)	NR	Y	NR	NR	NR	Y	NR
Blanchard, Mohan [15]	NR	NR	NR	NR	NR	NR	NR	NR
Borghei, Taghipour [32]	Cronbach α = 0.92 (total scale) Cronbach α is above 0.7 for all of the subscales.	NR	Y	Y	Y	Y	NR	NR
Cheung, Mok [33]	Cronbach α = 0.945	NR	NR	NR	NR	NR	NR	NR
Contreras-Yáñez, Ruiz-Medrano [20]	Cronbach’s α = 0.86(total scale)	Y	Y	Y	NR	Y	NR	NR
Corrigan [34]	NR	NR	NR	NR	NR	NR	NR	NR
Dempsey and Dunst [35]	Cronbach’s α = 0.93 (total scale)	NR	NR	NR	NR	Y	NR	NR
Diamond-Smith, Treleaven [36]	NR	NR	NR	NR	NR	NR	NR	NR
Farber and Maharaj [37]	Cronbach’s α is 0.80 and 0.82 (total scale) at pre- and posttests respectively	NR	NR	NR	NR	NR	NR	NR
Godoy, Patel [38]	NR	NR	NR	NR	NR	NR	NR	NR
Hansson and Björkman [39]	Cronbach α = 0.84 (total scale) Subscales: Cronbach α: 0.90 0.68 0.64 0.64 0.45 respectively	NR	NR	NR	NR	Y	NR	NR
Haswell, Kavanagh [17]	EES: Cronbach α = 0.891 12S: Cronbach α = 0.856	NR	NR	NR	NR	Y	NR	NR
Homko, Sivan [40]	Cronbach α = 0.94 (total scale)	Y	NR	NR	NR	NR	NR	NR
Jersky, Titmuss [13]	NR	NR	NR	NR	NR	NR	NR	NR
Kaczinski, Rosenheck [41]	Cronbach α:0.79, 0.82, 0.85 and 0.84, respectively at baseline, 1, 3 and 9 months (total scale)	Y	NR	NR	NR	Y	NR	NR
Kameda and Shimada [42]	Cronbach α = 0.99 (total scale) sub-scales ranged between 0.80 and 0.67	NR	NR	NR	Y	Y	NR	NR
Klima, Vonderheid [43]	English version: Cronbach α = 0.91 (total scale) Spanish version: Cronbach α = 0.93 (total scale)	NR	Y	NR	NR	Y	NR	NR
Koren, DeChillo [44]	Cronbach α ranged from 0.87 to 0.88	Y	Y	NR	NR	Y	NR	NR
LoGiudice, Josif [45]		NR	Y	NR	NR	NR	NR	NR
Patil, Klima [14]	Cronbach α > 0.95 (total scale)	NR	NR	NR	NR	NR	NR	NR
Nishita, Cardazone [46]	NR	NR	NR	NR	NR	NR	Y	NR
Yamada and Suzuki [47]	NR	NR	NR	NR	NR	NR	NR	NR
Castelein, van der Gaag [48]	ES: Cronbach α = 0.82 (total scale) mean inter-item correlation coefficient (MICC):0.14 Subscales: Cronbach α: 0.87;0.50;0.73;0.54 0.59 respectively PES: Cronbach α = 0.85 (total scale) MICC: 0.22 Subscales: Cronbach α = 0.85 0.77; 0.81 respectively MHCS: Cronbach α = 0.93 (total scale) MICC:0.45 Subscales: Cronbach α:0.85;0.88;0.87;0.76 respectively	NR	NR	NR	NR	Y	NR	NR
Bovill, Bar-Zeev [49]	NR	NR	NR	NR	NR	NR	NR	NR
Berry, Crowe [50]	NR	NR	NR	NR	NR	NR	Y	NR

*Y* Reported, *NR* Not reported

#### Validity

##### Content validity

The various methods of assessing content validity reported in six of the studies included brief descriptions of content revision [31, 45], calculation of the content validity ratio and content validity index [32], rating of measurement tool scale items by expert panels [20, 43], and independent item ratings and participants readability and clarity [44]. The face validity of measurement tools, for example, difficulty and relevance of response items, was assessed with a participant feedback approach in only two studies [20, 32].

##### Criterion-related validity

A comparative Locus of Control scale was used by Kameda and Shimada [42] to assess criterion-related validity in their development of an empowerment measurement tool for Japanese pregnant women. There was a strong positive correlation found between the original scale scores and the newly developed scale. Subsequently, in a 2015 study measuring empowerment among Iranian pregnant women, Borghei et al. [32] used Kameda’s pregnancy empowerment scale, as well as the Spritzer psychological empowerment scale as gold standards to evaluate the criterion-related validity of their new empowerment measurement tool (the Persian-language Self-Structured Pregnancy Empowerment Questionnaire), and showed a strong positive correlation between the gold standards and their new tool.

##### Construct validity

Construct validity was assessed by a number of different approaches in the studies in this review, including assessment of structural validity, internal and external construct validity, discriminant/convergent validity and cross-cultural validity. Structural validity was tested using an EFA method for determining number of factors of the scale in six studies. Klima et al. [43] used an expert panel to establish content validity of dimensions of pregnancy-related empowerment in an initial development phase of their empowerment measurement tool. A subsequent CFA was consistent with the expert panel’s four dimensions: provider connectedness, peer connectedness, skilful decision-making and gaining voice. Discriminant and convergent validity was assessed in two studies with fair results [41, 48]. Of five empowerment validation/translation studies, three considered an examination of cross-cultural validity. In developing a pregnancy-related empowerment scale, Klima et al. (2015) used a committee of bilingual translators to achieve conceptual rather than literal equivalence validation. Contreras-Yáñez et al. [20] conducted cultural sematic validation in a cross-cultural adaptation, and Hansson and Björkman [39] briefly mentioned cultural validation in the context of testing reliability and validity of the Swedish version of an English-language empowerment scale for people with a mental illness. Cross-cultural validity was not reported in the remaining two validation/translation studies [42, 48].

#### Responsiveness and interpretability

Responsiveness, or the ability of a measurement tool to detect changes over time, was examined in three studies [31, 46, 50]. Specifically, Nishita et al. [46] reported that a participant-driven management intervention enhanced diabetes self-efficacy with a medium to large effect size at follow-up after 12 months. Berry et al. [50] reported that effect sizes for four subscales of the Growth and Empowerment Measure (GEM) between baseline and 8 weeks were large, indicating that the GEM was sensitive to empowerment changes in the targeted substance abuse treatment population. Bhatta et al. [31] demonstrated sustained increased empowerment from a social self-value intervention for people with HIV after 6 months. None of the included studies reported interpretability.

#### Feasibility and clinical utility

Of the 26 studies reviewed, seven reported one or more aspects of measurement tool feasibility and/or clinical utility in terms of who carried out the assessment [15, 45], the number of missing responses [17, 20, 48], participants self-reported experiences of using the tool [20, 39, 43, 48], as well as the amount of time needed to complete an assessment [20, 39, 43].

Castelein et al. [48] in a comparison of three instruments, evaluated their clinical usefulness for people with psychotic disorders. They found grammatical and lexical considerations were important and that clinical usefulness was dependent on cognitive abilities of participants. Additionally, in feedback from participants, instrument items that were not applicable to all had the potential to confuse users during data collection and result in unanswered items [17, 48]. The average participant time needed was reported in three studies and ranged from 7 min to 30 min. Feedback related to the participant time burden showed that 7 min was regarded as convenient [20], whereas the 30-min timeframe required to complete the GEM [17] was considered too long for use with pregnant women in time-limited appointments with competing clinical priorities [49]. None of the studies reported whether staff training was provided ahead of measurement tool administration. Only Contreras-Yáñez et al. [20] assessed a majority of these features related to feasibility and clinical utility.

## Discussion

This systematic review has examined the measurement of empowerment in psychosocially vulnerable populations from 1990 to 2021. Since the early 1990s, empowerment as a general concept has gained significant appeal demonstrated by an exponential increase in literature, particularly that exploring its theoretical underpinnings [1]. The term is now entrenched among many of the health professions, however, over time efforts to develop robust empowerment measures have lagged [1, 44]. This review adds to this important field of enquiry by identifying empowerment measurement tools as they relate to psychosocially vulnerable populations, and reported on available assessments of psychometric properties of the tools, their feasibility and clinical utility.

Shortcomings in comprehensive testing of important measurement tool properties have been identified in the review. In assessments of reliability, or consistency of the measurement tools, most of the included studies appraised internal consistency as fair or good for the total scale making up the tool, but failed to assess or report on reliability of its subscales. Additionally, test-retest reliability or the degree to which results are repeatable has been reported as being a necessary testing component for adequately assessing general reliability [4], however, this step was documented in only four of the 26 included papers. Construct validity of a tool is one of the most significant measurement properties since it determines how well the tool measures what it claims to test [19]. Overall, this review identified a general lack of adequate investigation of this property with less than half of the studies (10/26) reporting results of an assessment.

With regard to five studies that included validation/translation, three examined cross-cultural validity, albeit one briefly, in the process of translating an existing empowerment tool to a new cultural and language group. Validating a tool in a culturally different population is not simply a matter of direct translation and back translation into respective language and cultural settings. Importantly, it is also necessary to ensure conceptual, operational, measurement, functional and item equivalence, in parallel with creating semantic equivalence [12, 51]. The application of standard scales without adequate adaptation inappropriately ‘presumes a universality of definition and understanding’ (Brown et al., 2013, p.6). For example, the pregnancy-related empowerment scale (PRES) was validated and widely used across America including African American populations [43]. However, for use in sub-Saharan African settings, translation was not considered and possible impact of cultural differences was absent in the study’s results [14]. Although there are varied available tools for assessing empowerment among pregnant women, it remains challenging to identify appropriate instruments that are applicable for the culture and experiences of each target population [43].

Responsiveness and interpretability of empowerment tools were described and reported in very few studies, which is consistent with findings of Terwee et al. [52]. Without insight about responsiveness, or ‘longitudinal validity’, it is difficult to understand whether clinically important changes in levels of participants’ empowerment are sustained over time. None of the studies included in this review tested interpretability which is useful in distinguishing clinically important change from measurement error. It is highlighted that responsiveness and interpretability, and floor/ceiling effect were often missing in measurement tool evaluations [19, 52]. Validation/translation studies could be more informative if they were able to test these important measurement qualities. Without full assessment of psychometric properties, the validity and reliability of results generated by use of that measure remain uncertain.

Most of the studies included in the review did not report enough information to assess feasibility and clinical utility of the empowerment tools. In particular, there was frequently a lack of information regarding time and effort needed for participants to complete assessments, or for those who administer them. Measurement tool evaluations should also provide an indication of training or professional expertise and experience needed by staff who administer instruments. As matters of practicality, decisions based on the respondent and administrative burden of a measurement tool are likely to be linked to available resources in both clinical and research environments. Additional instrument attributes related to feasibility of use and clinical utility include the needed literacy levels of intended participants and user acceptability [53]. High participant refusal rates and levels of missing data are probable indicators that an instrument or items in it were unacceptable or not applicable. Missing responses are particularly important for clinical utility if the total score from an empowerment measurement tool is influenced by unanswered items [25].

Whilst some empowerment scales have been successfully validated across populations, settings and cultures, they may not measure up in a cursory assessment of their feasibility or clinical application. For example, the GEM was developed and validated with Aboriginal Australians and studies have reported that it effectively captured changes within Indigenous people participating in specific empowerment programs [13, 17, 45]. The GEM requires significant investment for implementation as it encourages participants to reflect on their life experiences and requires an average of 30 min to complete the scale [49]. Empowerment is inherently complex and subjective, context dependent, and definitionally imprecise [17]. As such, it could be argued that as a construct regarded with increasing importance and value, its measurement is deserving of additional participant and administrative burden. Although a shortened version of the GEM reducing the instrument from 12 to six core item scales has been trialed and successfully detected the most consistent empowerment change in two groups of participants [54], it was concluded that using the full tool gave maximum analytical power for understanding the nuances of personal change. Development and rigorous validation of short-form scales may enhance the routine use of empowerment measurement tools [55], however, the advantages of this should be weighed against potential loss of intent and utility of the original tools.

As with many literature reviews, relevant articles may have been missed by our search strategy or overlooked in error during the title and abstract review phase. It is possible that an important but unpublished body of work related to empowerment of psychosocially vulnerable populations exists. For example, projects undertaken in Indigenous community-controlled sectors internationally may be underreported in the peer-reviewed literature. This review is also subject to potential bias including errors in translation of information from original research papers. Due to the time lag between research completion and subsequent publication recent literature may have been missed. A further possible bias was introduced because this review has excluded literature published in languages other than English.

## Conclusion

This review synthesizes and assesses available studies on the measurement properties, feasibility, and clinical utility of empowerment measurement tools used in psychosocially vulnerable populations. Few studies provided a comprehensive assessment of the properties of interest. There were significant shortcomings in testing of psychometric qualities, particularly with regard to evidence to support responsiveness and interpretability of the measurement tools. The results highlight that development, translation and validation of empowerment measurement tools is not a straightforward process [56]. There are many steps that can be costly, time consuming and requiring complex statistical analyses. Nevertheless, the work is important because well-designed and tested measurement tools are fundamental to increasing our understanding of the complex empowerment construct. Detailed and importantly, systematic assessments of the psychometric properties of measurement tools are needed to create reliable, valid and responsive measures of empowerment. Additionally, future research will benefit from including feasibility and clinical utility as outcome measures in assessments of the effectiveness of empowerment programs for psychosocially vulnerable populations.

## Data Availability

Not applicable.
